# Supplementary material for: Analysis of a Casimir-driven parametric amplifier with resilience to Casimir pull-in for MEMS single-point magnetic gradiometry
Source: Microsyst Nanoeng. 2021 Sep 7;7:73. doi: 10.1038/s41378-021-00289-4 (PMC8433440; doi:10.1038/s41378-021-00289-4)
Supplement: Supplementary file 1 — Supplementary Material [file 41378_2021_289_MOESM1_ESM.docx]

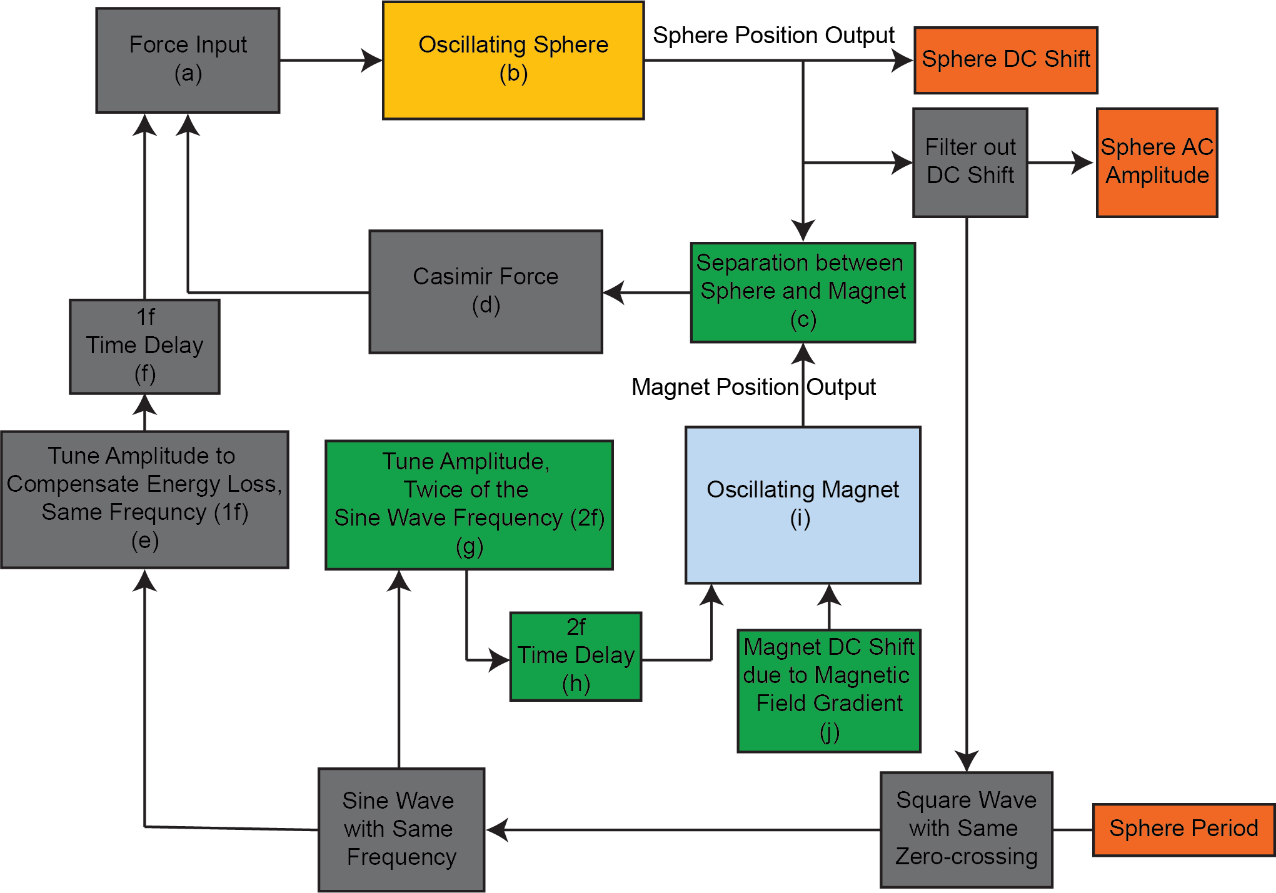


**Fig. S1**. **Detailed Schematic of the Casimir Gradiometer Simulation**. The simulation is conducted using a combination of Simulink, Simscape, and MATLAB. Two resonators are set to dynamically interact, parametrically modulated by the Casimir Force. Outputs are shown in orange. The magnet (blue), sphere (yellow), conditioning (gray and green) are also shown.

In our simulation, block (b) is the oscillating sphere with 1 kHz nature frequency and a quality factor of 1000. A low pass filter is used to optionally monitor the DC shift in the sphere position. The AC sphere position is also conditioned by a high pass filter (10 Hz cut-off) to measure the AC amplitude. The zero-crossing of this filtered signal (negative slope) is detected, cast to a Boolean variable, and processed by a JK flip-flop block to generate a square wave in phase with the sphere oscillation. The square wave amplitude is 1 and the period is recorded. The square wave, in turn, is used to generate sine wave at f and at 2f. These signals propagate through the 1f time delay block (f) and 2f time delay block (h), respectively. This method is chosen as it can respond dynamically to an arbitrary waveform. Simulink has a variable phase shift block, but it cannot be tuned during simulation to a changing input. The amplitude and phase of the 1f signal is conditioned (e) such that the sphere’s amplitude does not decay due to damping, a method which has been described and experimentally implemented earlier [1]. The 2f signal, which has twice the frequency of the sphere, imposes a force on the magnet with tunable amplitude and delay (g). A second force (j), from a gradient magnetic field, is optionally applied. This force can produce a static shift, or can be a dynamic signal, as is discussed in the text. The summation of sphere position, magnet position, and tunable initial separation (c) is the real-time separation. The actual separation is used to calculate the contribution from the Casimir Force (d) on the sphere. The tuned 1f signal and Casimir force sum to generate the force input (a) and feed back to the sphere system. Therefore, by adjusting different 2f amplitude, time delay, initial separation and DC shift of magnet, one can comprehend how the sphere AC amplitude, DC shift and period (or frequency) evolve with time for parameter values in the simulation setting.

[1] Barrett, Lawrence & Imboden, Matthias & Javor, Josh & Campbell, David & Bishop, David. (2020). Modal Engineering for MEMS Devices: Application to Galvos and Scanners. 10.31224/osf.io/n53fx.
